# King Kong Pox: A Case of Severe Mpox and Bartonella Co-infection in a Patient Living With Human Immunodeficiency Virus (HIV)

**DOI:** 10.7759/cureus.94219

**Published:** 2025-10-09

**Authors:** Anurag Karki, Ashirbad Acharya, Suraj Shrestha, Puja Thapa, Yogesh Pandey, Ricardo Conti, Lindsay Lim

**Affiliations:** 1 Internal Medicine, AdventHealth Orlando, Orlando, USA; 2 Internal Medicine, Tribhuvan University, Kirtipur, NPL; 3 Health Administration, University of Central Florida, Orlando, USA; 4 Infectious Disease, AdventHealth Orlando, Orlando, USA

**Keywords:** brincidofovir, hiv/aids, immune reconstitution inflammatory syndrome (iris), mpox, refractory mpox, tecovirimat, vaccinia immunoglobulin

## Abstract

Mpox, formerly monkeypox (MPXV), is a zoonotic virus that has emerged globally in recent times, disproportionately affecting immunocompromised individuals, particularly people living with human immunodeficiency virus (HIV) (PWH). PWH with advanced HIV and low CD4 counts are at higher risk for severe, prolonged courses and sometimes fatal Mpox infections, which may often be complicated by bacterial superinfections and opportunistic co-infections. We present a case of Mpox in a PWH with an initial CD4 count of 2 cells/μL and a high HIV viral load, who had worsening disease despite treatment with tecovirimat, raising the question of persistent/resistant Mpox. Further investigations revealed co-infections with *Bartonella* and *Cytomegalovirus* (CMV), adding to the case's complexity. He initially presented with high-grade fever, and multiple, large necrotic lesions were noted on his face, scalp, left hand and knee, back, rectum, and genitalia. The patient was initiated on treatment with IV tecovirimat, oral brincidofovir, and vaccinia immunoglobulin (VIGIV). Anti-retroviral therapy (ART) was rapidly re-initiated. His hospital stay was further complicated by a superimposed extended-spectrum beta-lactamase (ESBL) *Escherichia coli* abscess underlying his necrotic left knee lesion. While admitted, he completed a two-week course of IV tecovirimat, three doses of brincidofovir, and a single dose of VIGIV with gradual improvement and no new lesions. This case demonstrates the potential for Mpox to have a prolonged and extensive course with widespread necrotic ulcers in the setting of HIV despite treatment. It is critical to have timely diagnosis and treatment of both Mpox and underlying HIV, alongside management of co-infections for improving disease outcomes.

## Introduction

Mpox (formerly monkeypox) virus (MPXV) is a zoonotic disease historically confined to regions of Africa, with limited human-to-human transmission before 2022 [1,2]. The 2022-2023 global outbreaks marked a shift, with over 90% of confirmed MPXV cases occurring among gay, bisexual, and other men who have sex with men (MSM), a population at high risk for both MPXV and human immunodeficiency virus (HIV) [3]. People living with HIV (PWH), specifically those with CD4 T-cell counts less than 100 cells/μL and untreated advanced HIV, are disproportionately affected, which includes around 40-50% of Mpox cases, and they experience more prolonged and potentially lethal disease [4-6]. They also often present with bigger skin lesions, bacterial superinfections, and protracted illness [7,8]. Co-infections with opportunistic pathogens, such as *Bartonella *and *Cytomegalovirus *(CMV), further complicate the clinical course, especially in severely immunocompromised patients not on anti-retroviral therapy (ART) [5,6].

The following case of PWH with worsening Mpox despite treatment highlights a few challenges, such as persistent/resistant MPXV, opportunistic co-infections with *Bartonella* and CMV, and possible immune reconstitution inflammatory syndrome (IRIS).

## Case presentation

**Figure 1:**
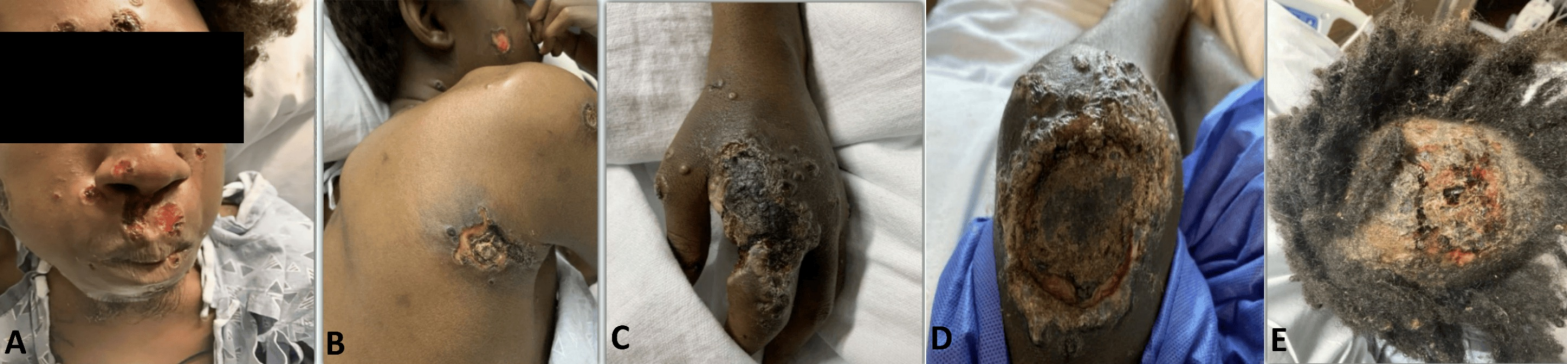
Disseminated ulcerative and necrotic skin lesions. (A) Facial involvement with multiple ulcerative nodules, especially around the nose and mouth, with presence of hemorrhagic crusts. (B) Well-defined ulcers, core necrosis, and surrounding erythema on the posterior shoulder. (C) A large ulcer with black eschar and surrounding pustules on the dorsum of the hand. (D) Lesion on the left knee with central necrosis and a thick hyperkeratotic border. (E) Scalp ulcer with hemorrhagic crusts and necrotic tissue on top.

A 24-year-old African American MSM, living with HIV but not adherent to ART, presented to the emergency department (ED) of our hospital with worsening Mpox lesions. He was initially diagnosed with Mpox via polymerase chain reaction (PCR) two months prior at another facility where he had presented with small necrotic lesions on his hands and face and had completed a two 14-day course of tecovirimat. Despite his initial therapies, his condition had deteriorated with new lesions and high-grade fevers with occasional anal bleeding, prompting presentation to our hospital. On examination, he had multiple large, necrotic lesions on his face, scalp, left hand, knee, back, rectum, and genitalia, some of which are shown in Figure 1.

**Figure 2:**
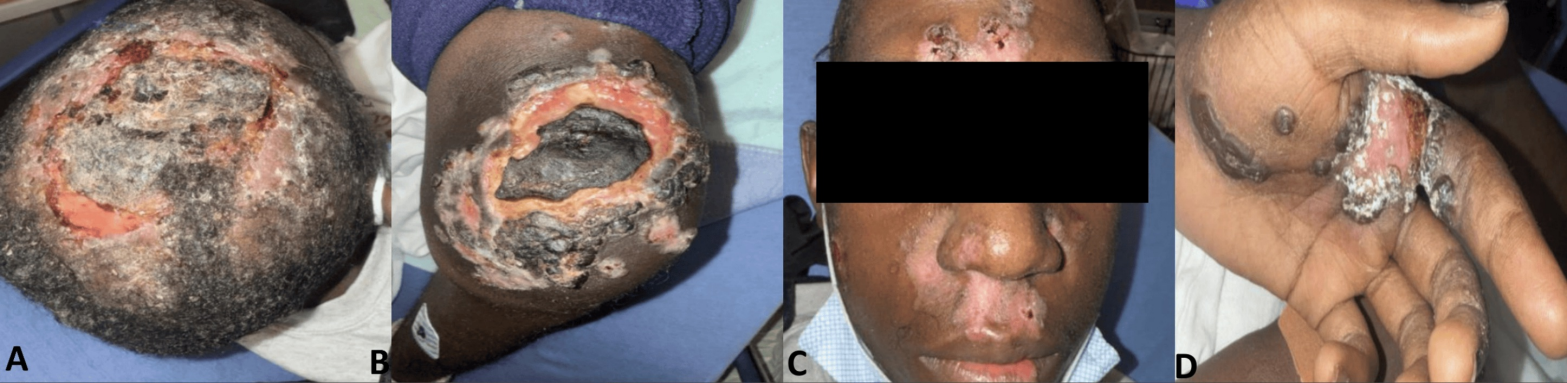
Improvements of Mpox lesions in various anatomical regions throughout time. (A) Scalp lesions exhibiting central crusting and resolving erythema at the periphery with partial re-epithelialization. (B) Desquamation and granulation tissue showing signs of healing in this ulcerated necrotic lesion on the left knee. (C) Facial lesions showing resolving erythematous plaques and crusting over the forehead and nasal bridge. (D) Left hand palmar aspect showing a healing ulcer with dry crusts and granulating base.

Repeated MPXV PCR tests confirmed persistent infection. His initial CD4 count was 2 cells/μL (normal range: 500-1600 cells/μL), with an HIV viral load of 687,000 copies/mL. Due to the severity of his condition, the case was discussed with the Centers for Disease Control and Prevention (CDC). After completing a two-week course of IV tecovirimat, three doses of brincidofovir, and a single dose of vaccinia immunoglobulin (VIGIV), the patient showed gradual improvement with no new lesions. ART was initiated during his prior admission, but he was unable to continue it as an outpatient. ART was reintroduced. His initial CD4+ T-cell count of only 2 cells/μL and a significantly high HIV-1 viral load of 687,000 copies/mL indicated the patient's severe immunosuppression. Considering the severity of immunocompromised status, a thorough assessment for opportunistic infections was done. He had *Bartonella henselae* serology positive for IgG with a titer of 1:256 (normal range: less than 1:128) and IgM with a titer of 1:64 (normal range: less than 1:16), suggesting either a recent or chronic infection. The patient had also reported exposure to stray cats in the past. CMV DNA was also found in the blood with a viral load of 5,280 copies/mL (normally undetectable), which is also a prevalent opportunistic infection in individuals with advanced acquired immunodeficiency syndrome (AIDS). Treatment for both infections was initiated with appropriate antibiotics that included doxycycline and valganciclovir. His hospital course was further complicated by an extended-spectrum beta-lactamase (ESBL) *Escherichia coli* abscess underlying a necrotic knee lesion. After completing a two-week course of IV tecovirimat 200 mg twice daily, three doses of oral brincidofovir 200 mg, and a single dose of VIGIV 6000 units, the patient showed gradual improvement with no new lesions as demonstrated in Figure 2. He was discharged with a four-week course of oral tecovirimat 600 mg twice daily and outpatient follow-up.

## Discussion

The Mpox outbreak in 2022 found sexual contact as the primary route of transmission, with frequent anogenital lesions and co-occurring sexually transmitted infections (STIs) such as gonorrhea, syphilis, and chlamydia [4,5]. MPXV infects humans through mucosal or skin contact, leading to primary infections in the eyes, respiratory system, skin, or genital sites [9]. The virus can disseminate via lymphatic and vascular routes, causing lymphadenopathy and affecting organs such as the lungs, liver, and kidneys [9,10]. Bacterial superinfections, co-infections with HIV or STIs, worsen disease severity, prolong recovery, and increase morbidity and mortality [11]. 

While initial prodromal symptoms of Mpox are similar in PWH and those without HIV, PWH with advanced HIV and uncontrolled viremia experience more severe and prolonged cutaneous manifestations, often with mucosal involvement [5,12]. Compared to HIV-negative individuals, PWH are more likely to develop ano-rectal pain, bleeding, proctitis, peri-rectal abscesses, and phimosis, with advanced HIV increasing the risk of multi-organ involvement, including myopericarditis, encephalitis, colitis, pneumonitis, blepharoconjunctivitis, and keratitis [9,10,13]. In PWH with CD4 counts below 350 cells/μL, Mpox severity increases with declining immune function, particularly in those newly diagnosed with advanced HIV or not on ART [6,13,14]. A study in Atlanta found that PWH with uncontrolled HIV viral loads (>200 copies/mL) had 2.1 times higher odds of severe Mpox compared to those with suppressed viral loads [15]. Our patient had an exceptionally low CD4 count with a high viral load that had placed him at the highest risk for a prolonged and severe Mpox disease course. *Bartonella henselae* can also have widespread skin manifestations in advanced cases of HIV, most notably bacillary angiomatosis [16]. Our patient had positive serologies for *Bartonella* that were consistent with acute/recent infection; however, biopsy and *Bartonella* PCR from select lesions were negative. Workup was limited due to the sheer number of lesions, making it more difficult to definitively rule out bacillary angiomatosis. This co-infection had likely contributed to the initial treatment failure and prolonged course, which emphasizes the necessity of a comprehensive diagnostic workup for co-pathogens in severely immunocompromised Mpox patients.

No MPXV-specific antivirals are approved, but tecovirimat and brincidofovir, developed for smallpox, are used for Mpox treatment [17]. Tecovirimat has been the first-line therapy, with observational studies showing no significant difference in outcomes by HIV status [18,19]. Brincidofovir, a prodrug of cidofovir, may act synergistically with tecovirimat in severe cases, particularly in PWH with advanced HIV [20]. VIGIV provides cross-protective antibodies and may benefit immunocompromised patients [21]. In our case, we had to use all of the above three modalities, which then potentially led to improvement in his overall clinical status. This aggressive approach was ultimately necessary and beneficial in this context of severe immunosuppression and initial treatment failure.

Initiating ART can trigger Mpox-IRIS in PWH with advanced HIV, with 25% of such patients in a global case series developing suspected IRIS and a 57% mortality rate among those affected [5]. Despite this risk, early ART initiation is critical for immune reconstitution and MPXV clearance. Combination antiviral therapy is recommended for severe, progressive disease in such patients [22]. Persistent or recurrent Mpox in immunocompromised patients may indicate tecovirimat resistance, with studies identifying resistant isolates in patients with CD4 counts below 200 cells/μL who received multiple tecovirimat courses [5]. We had initial concerns for Mpox-IRIS, but ART was continued in this patient. 

## Conclusions

Mpox is an opportunistic infection that can cause severe disease in PWH, particularly those with advanced HIV, along with low CD4 counts. Timely diagnosis and treatment of both Mpox and underlying HIV, alongside management of co-infections, are critical for improving outcomes. Early ART initiation, antiviral therapy, and supportive care are essential, despite risks such as IRIS or antiviral resistance. Comprehensive care and vigilant monitoring can mitigate the severe manifestations of Mpox in this vulnerable population.
